# When xenotransplantation enters the conversation: reflections on public involvement in research on a novel and controversial technology

**DOI:** 10.1186/s40900-026-00894-5

**Published:** 2026-05-04

**Authors:** Agata Pacho, Clara Martins de Barros, Carole Lamouline, Mustafa Al-Haboubi, Paul Boadu, Nicholas Mays

**Affiliations:** 1 Policy Innovation and Evaluation Research Unit, Department of Health Services Research & Policy, London School of Hygiene & Tropical Medicine, 15–17 Tavistock Place, London, WC1H 9SH UK; 2 Department of Sociology, Purdue University, Beering Hall Suite 1144, West Lafayette, IN 47907 USA; 3 Public contributor, NHS National Health Service, London, UK

**Keywords:** PPIE, Public involvement, Novel technology, Transplantation, Xenotransplantation, Research ethics

## Abstract

**Background:**

Xenotransplantation (XT) is being explored as a potential solution to organ shortages. Driven by revived scientific interest, XT has sparked debates that remain ethically charged. While much literature focuses on why community engagement is needed, this paper examines how XT itself reshapes the public involvement. We reflect on collaboration between researchers and public contributors during development of a national survey on UK public attitudes towards XT. Co-written by researchers and contributors, the paper draws on meeting notes and email exchanges to examine how XT shaped group dynamics.

**Main body:**

We argue that XT is not only a biomedical innovation but also a disruptive force in public involvement in research in three ways. First, as a distant prospect, it permits asking philosophical rather than merely practical questions. Second, its scientific uncertainty may unsettle the expert–public divide, fostering shared curiosity and speculation. Third, XT’s emergent status makes visible how research may shape what XT becomes. Working on the development of the survey, we were thus not only engaging with XT’s content but also reflecting on their role in its unfolding. Our reflections highlight how focus on technologies that remain in exploratory phases can reshape researcher–contributor dynamics by flattening hierarchies. While often framed through technological determinism, XT was instead experienced as contingent, something open to questioning, negotiation, and resistance. Rather than being the sole domain of experts, it appeared amenable to influence through research and public involvement.

**Conclusion:**

XT, being a novel and controversial technology, unsettles conventional models of public involvement in research, opening new possibilities for participation and challenging assumptions about its purpose and dynamics.

**Supplementary Information:**

The online version contains supplementary material available at 10.1186/s40900-026-00894-5.

## Abstract (plain language summary)

**Background:**

There is a persistent global shortage of organs for transplantation that leads to long transplant waiting lists and thousands of deaths each year. One potential solution to this shortage being explored is xenotransplantation (XT), which is the transplantation of living cells, tissues, or organs from animals into humans. Current research focuses on the use of genetically engineered kidneys and hearts from pigs for transplantation. Here, we reflect on collaboration between researchers and public contributors during development of a national survey on UK public attitudes towards XT.

**Main body:**

We argue that the topic of XT, by being novel and controversial, can reshape familiar dynamics of public involvement and open up new avenues for participation. Firstly, in our experience, the topic gave an opportunity for public contributors to steer the conversation away from the more practical matters (e.g., wording of the survey) to address philosophical questions (e.g. whether humans have the right to use animals to extend human life). Secondly, because of the scientific uncertainty surrounding XT, researchers felt often out of their depth. This disrupted, the often-felt in public involvement, expert–public divide. Thirdly, the novelty of XT, made it apparent how research might shape what XT becomes. Therefore, public contributors were not only engaging with the content of the survey but played a role in establishing what XT was becoming.

**Conclusion:**

XT unsettles conventional ways of public involvement in research. It may open new possibilities for participation and challenge assumptions about the purpose and dynamics of public involvement.

**Supplementary Information:**

The online version contains supplementary material available at 10.1186/s40900-026-00894-5.

## Background

There is a persistent global shortage of organs for transplantation. Demand continues to exceed the limited supply of suitable organs, which results in long waiting lists and thousands of deaths each year [1]. One potential solution to this shortage being explored is xenotransplantation (XT), in particular the transplantation of whole organs from non-human animals into humans. Currently, research focuses on developing the clinical potential of genetically engineered kidneys and hearts from pigs specifically raised for that purpose and kept in sterile conditions [2]. In February 2025, the US Food and Drug Administration (FDA) approved the first clinical trial testing whether porcine kidneys could be safely transplanted into patients with end-stage kidney disease [3]. This follows a small number of high-profile kidney and heart xenotransplants involving living patients in the United States (US) in recent years.

These advances, while bringing the field closer to clinical reality, have also renewed a debate on the ethics, safety, and societal implications of XT [4]. Public debate and interest in the field was first ignited in the 1980s when an American infant, known as “Baby Fae”, received a baboon heart. While the transplant ultimately failed, the event raised questions about informed consent, the morality of killing an animal to save a human life and the governance of experimental medicine involving humans [5]. Since then, XT has periodically reemerged in public discourse, remaining a source of controversy and raising ethical challenges. This is primarily because, while any organ transplantation unsettles traditional dichotomies of self and others, XT also disturbs the boundaries between human and animal [6–8]. Consequently, those working in the field have advocated for and organised community engagement [6, 9–11] showing the diversity of opinions and attitudes and how the public raise new questions for debate on the ethics of XT [12].

While much of the literature has focused on *why* the public needs to be included in the debates on the future of XT and public’s perceptions of XT, this paper shifts the focus to *how* XT itself reshapes public involvement in research. Uneven power dynamics between researchers and public contributors have previously been identified as a significant challenge to meaningful and ethical public involvement in research [13–15]. Such hierarchies are especially difficult to challenge when researchers work within funding and time constraints that limit opportunities for more mindful engagement with public contributors. We propose that XT, as a biomedical innovation that remains under exploration rather than routine clinical application, and for which key questions of safety and efficacy persist, may be a disruptive force within the landscape of public involvement in research. By that, we mean that it opens up new possibilities for participation and unsettles the more conventional dynamics of public involvement in research in policy research.

We draw on our experiences of collaborating as academics and public contributors on carrying out a national survey to explore UK public attitudes towards XT. The survey was conducted as a response to a request by the XT Expert Subgroup of its Implementation Steering Group on Organ Utilisation (ISOU). The XT Expert Subgroup was established by the Department of Health and Social Care (DHSC) to provide preliminary advice on the feasibility of XT within the National Health Service (NHS) and on the issues that will need to be resolved should it be adopted as a treatment option.

The survey included perceptions of acceptability of XT, perceived benefits and risks, and how these views vary across different segments of the population. The work was to be completed within a 10-month timeframe. While the survey findings have been reported elsewhere [16], this paper offers reflections on the ways in which the researchers and public contributors worked together on the development of the study questionnaire.

## Who we are

This essay is written by Policy Research Unit in Policy Innovation and Evaluation (PIRU) researchers (AP, MAH, PB, NM) and two out of five public contributors who supported the study (CMDB, CL). PIRU is an independent research centre, funded by the National Institute for Health and Care Research (NIHR), who undertake projects designed to contribute to the evidence-informed advice provided to the civil servants in UK Government Departments and arm’s length agencies on health improvement, health care, and social care policies.

At PIRU, Patient and Public Involvement and Engagement (PPIE) is a core element of research activities. Our strategy holds that public contributors ought to be involved in research from the early stages enabling them to shape research questions and methods and to remain engaged through to dissemination of results [17]. Yet, as policy researchers, we often face constraints related to tight timelines and budgets. The research projects typically run for 12 to 24 months and are commissioned in relation to the needs and priorities of Ministers in the DHSC. These factors can pose challenges to meaningful PPIE limiting opportunities to revisit and refine engagement strategies, build deeper connections with PPIE contributors, or fully explore complex issues within the scope of each project. Here, we show how introducing XT as a topic of conversation with public contributors, reshaped familiar dynamics and opened up new avenues for participation, addressing certain challenges while introducing new ones. We propose that uncertain and somewhat controversial issues like XT create greater opportunities for rich debate between policy researchers and public collaborators than more established health care issues.

## Public contributors

Public contributors were recruited via existing PIRU networks and through advertising on the People in Research platform (https://www.peopleinresearch.org/), an online board promoting public involvement opportunities. As the research on XT differed from other studies conducted by PIRU, it necessitated taking a different approach to recruitment of the public contributors. Our research focuses on the impacts of policy, and we do it for those who implement it and those whose lives are affected by it. Because of this responsiveness to the policymakers and the urgency of many projects, we often work with members of the public who have personal experience of particular health conditions or challenges in the health and social care sector that relate directly to the policy in question. The expectation of the collaboration with the public is that it may influence the sector. Unlike most of the PIRU’s work, this project did not aim to evaluate an established policy or intervention. Rather, it sought to explore public perspectives on an intervention that may be introduced into the UK health system in the future. Subsequently, the advertisement for public contributors to join the XT project PPIE group, invited people with an interest in the topic rather than any relevant personal experience as occasionally happens in PPIE work but is not the norm. Yet, as we go on to show in this paper, those who participated did so because they felt a degree of proximity to the issue; for example, through recalling a family member who had undergone surgery involving a porcine heart valve, or through holding worldviews that were at odds with XT. Five public contributors joined the project’s PPIE group, with four identifying as women and one identifying as a man. The contributors came from different ethnic and religious backgrounds and migration history. While public contributors occasionally drew on their personal backgrounds to contextualise their views on XT, they were not expected to speak on behalf of their communities, nor were their contributions treated as representative in that sense.

Between March and November 2024, public contributors participated in four online meetings held on Zoom. All public contributors had extensive experience participating in online PPIE meetings. While holding meetings on Zoom has helped make participation more accessible, particularly for contributors with disabilities, the lack of in-person interaction meant it took longer to establish familiarity. In addition, technical issues occasionally created friction and misunderstandings, for example when a participant was inadvertently disconnected and cut off mid-sentence without realising it. Although the initial meeting was used to build rapport before moving on to discussions of XT, those of us who had previously worked together on other projects experienced these interactions as a process of reconnection, felt more natural and free-flowing.

The first meeting served as an introduction to the research and the topic of XT. Public contributors were offered information on the most recent scientific developments in XT, its potential to address the shortfall in human organs for transplantation, associated risks (such as zoonotic infection), and the conditions under which pigs would be reared. While we focused on the use of whole organs for transplantation, we referred to forms of XT that are already present in clinical practice, such as the use of porcine heart valves. The second and third meetings were used to gather input on survey content and wording, aiming to improve clarity and relevance. The final meeting was dedicated to discussing the survey findings and reflecting on the project. Throughout the study, public contributors guided us on which topics to explore in the survey, with concerns about animal welfare emerging as the most pressing. They also reviewed drafts of the survey to improve clarity and understanding, co-developed short pieces of information about XT that were included at intervals in the survey and suggested directions for future research. The final version of the survey can be viewed in the Appendix where we highlighted the key contributions made by the public contributors.

In this paper, we will predominantly focus on the work that we did as researchers and public contributors together prior to the survey rollout. The reflections below were written based on notes taken during the meetings and email exchanges in-between the meetings. Handwritten notes were taken by AP, who led the PIRU public involvement activities, organised the meetings, and maintained contact with public contributors between sessions. Summaries of these notes were shared via email with both researchers and public contributors between meetings. AP also used the notes reflexively to consider the researchers’ experiences of the meetings, particularly the growing curiosity about how discussions with public contributors revealed XT as a controversial issue and challenged the underlying premises of the research as well as the choice of methods. The four sets of notes (one per meeting) included two email exchanges between AP and two public contributors. In these emails, contributors reiterated what they had said during the meetings and were therefore treated as a continuation of the discussions.

While we will share the views of the public contributors on XT where relevant, we are predominantly interested in the process of public involvement in research on a novel and controversial biomedical technology. We reflect on how this work differed from other public involvement work we have experience of as policy researchers to show how the XT itself, being novel, controversial and not yet fully understood, affected how we worked, the dynamics within the group and particular tensions that arose throughout.

## Writing up our reflections

The initial draft of this paper was prepared by AP who used her sets of notes to reflect on the process of working with public contributors and, as a result, outline the three main ways in which the conversations with public contributors about XT differed from conversations with similar panels established for other studies: opportunity to wonder, disrupting hierarchies and making XT. Those served as a starting point for exploring the richness of these interactions. AP then shared the draft with the other researchers (MAH, PB, NM) as well as the public contributors, who were invited to edit, comment on, and contribute to the next version by adding their reflections on the process of working on the questionnaire. One public contributor provided editorial input, while two others (CMDB and CL) joined as co-authors. As their reflections emerged as distinctive contributions in their own right, the authors decided to present them in boxed sections throughout this article, situating them in dialogue with the main text and highlighting the role of the public contributors.

AP is an early career researcher and a social scientist interested in social and cultural aspects of health and health care delivery and public involvement lead for PIRU. She had not worked in research on organ transplantation prior to this study and had no relevant personal experiences. Drawn to the novelty of XT, she hoped to explore the ethics of it.

CMDB and CL bring extensive experience as patient and public contributors in research. At the same time, they found the topic of XT novel and, like AP, did not have prior in-depth knowledge of it. Rather, they expressed interest in the science as well as the moral and societal questions that arise when the boundaries between human and animal life are redrawn by new technologies.

MAH is a mixed methods’ researcher who had previously worked on an evaluation of the implementation of the changes to the organ donation law (Deemed Consent) in England. In that project, he led on two surveys to that sought to establish the views of health professionals in England on the changes to the law. He therefore had no prior experience of researching public views on organ transplantation or XT, which is what drew him to this research.

PB is an Economist who had previously worked on an evaluation of the implementation of the changes to the organ donation law (Deemed Consent) in England, where he led in the analysis of Organ Donation Attitudinal Tracker survey dataset collected by National Health Services Blood and Transplant to assess public attitudes, preferences and self-reported behaviour related to deceased organ donation. He also led in a study that assessed public intentions to become a living kidney donor in England. He has some experience in public engagement in research in other fields, other than health and social care.

NM is a senior researcher and former Director of PIRU with long experience of policy-related health and care research, including social surveys. He too was drawn to the topic by its novelty, and the ethical and philosophical issues it raised. He had proposed research on public, patient and professional views on XT to the DHSC just as the XT Expert Sub-Group was making its request for research.

While none of the co-authors had in-depth technical knowledge of XT, the research team included a clinician with expertise in nephrology and transplantation, though not directly involved in XT, who joined the final meeting with public contributors. While we sought feedback from the XT Expert Sub-Group on the design of the survey to ensure its’ alignment with the scientific knowledge of XT, they were not present during our meetings with public contributors. This allowed greater independence for researchers in conducting the study and opened space for more critical engagement with scientific developments in meetings with public contributors.

The work on which we reflect here took place in the UK, where public involvement in health research has been promoted and formalised since the 1990s [18]. Despite these efforts, literature points to persistent shortcomings, including limited inclusiveness, accessibility, and tokenism [19]. Our reflections may not be generalisable to all settings, particularly those with different approaches to public involvement.

## Opportunity to wonder

**Table:** 

I was one of the PPIE members on the project set up to review the questionnaire about XT and I found it so exciting, even fascinating, rewarding and refreshing: mainly because it was different, different from the more “traditional” work that PPIE panels are usually engaged in. Traditionally, we’re discussing how well research study is going, how relevant the research work is to patient and members of the public. We contribute to it by bringing our own perspectives and lived experience to ensure our voices are heard and represented, or we may review documents. In this project, our engagement was meant to assess the relevance and accessibility of a survey aiming to understand public’s perception of XT. That happened; but much more did too! We brainstormed a great deal about XT of course, and its implications, but also about religion, faith, beliefs, ethics and philosophy. It was interesting to notice the wide variety of reactions
from members of the group. Everybody had their own, slightly different point of view. We were quite thorough at analysing what accepting and putting XT into practice could do to our interactions with animal world and how the understanding and respect humans currently have for animals as well as some ethical barriers may then be compromised. (CL)

It may be years before our research on XT influences actual policy. And since the study was not responding to a clearly established policy need due to XT not being applied routinely as a viable transplant option yet, despite its short 10-month timeline, it did not carry a sense of urgency. This lack of urgency, combined with the absence of experience or expertise in the field to draw on, created a distinctive space between the subject of the research and us, opening up an opportunity to wonder. Although we structured the meetings around the practical steps of questionnaire development, discussions in each session often shifted towards philosophical questions, such as whether humans have the right to use animals to extend human life. This, in turn, led to moments of tension between public contributors with differing worldviews, reflecting wider social debates around XT.

**Table:** 

There were moments during our meetings when the conversations moved away from the task at hand (refining the survey) and toward deeper moral, cultural, and even spiritual discussions. I worried at times that we were going off track, but I now think those digressions were essential. They allowed us to locate ourselves in the conversation, to speak from our lived realities. For me,
that’s where true involvement happens, not just in answering researchers’ questions, but in helping to shape what questions get asked in the first place. (CMDB)

The dominant argument in favour of XT is often framed in terms of the moral imperative and a collective obligation to do whatever is possible to save human lives and alleviate human suffering [20]. In contrast, ethical objections to XT often raise animal welfare concerns [21, 22]. Throughout the meetings, some public contributors remained critical of the assumption underlying XT that humans have the right to kill animals in order to preserve human life. They challenged reassurances provided by some of the XT Expert Subgroup members regarding the welfare of pigs bred specifically for XT. In particular, some questioned whether claims about high-quality living environments for these animals were based on an anthropocentric view, which fails to consider what pigs themselves might need. As one public contributor remarked, pigs may not value indoor comfort as humans do. Instead, they might simply want to be outside which would not be possible to prevent exposure to infection. The conversations often turned to broader ethical implications, particularly the idea that XT reinforces a hierarchy of life, with human lives placed above all others. One public contributor expressed a worry about the wider social impact of this assumption:

*I wonder if we should ask, if treating animals as a commodity has an effect on the wellbeing of society, particularly growing children? Will it breed a generation of harsh, unkind and cruel people?*

Another public contributor expressed a concern about what it meant to collaborate with US-based companies, which she saw as both, very profitable and contributing to health inequities:

*If there is funding for this*,* and it involves the USA*,* why not utilise available funding for our national benefit without involving a country where health is hugely privatised and a lot of private companies are making billions? (…) US companies already have billions of profits whilst having the worst health inequalities in the world*.

While the public contributors were invited to engage in the practical tasks of developing and testing the questionnaire, the conversations often moved beyond the practical to explore philosophical questions surrounding XT such as how it reshapes our relationship with non-human animals and the planet. Their reflections highlighted a deeper concern about the interplay of biomedical innovation, social values, morals, and our collective future. Specifically, they echoed the concept of co-evolution between technology and society [23], reflecting the broader argument that scientific innovation not only produces an intervention but also engenders the environment within which said intervention operates [24, 25]. Although we have suggested earlier that the study lacked urgency in the sense of having to contribute to existing or imminent policy decisions, the way we wondered together carried a different kind of urgency - one rooted in questions of life and our responsibilities towards the planet. In other words, the urgency was not inscribed in the project itself or in how it was presented to the public contributors; rather, it emerged in response to the object of our interest, the XT, and through the wondering prompted by the public contributors’ reflections.

**Table:** 

From the outset, it was clear that this project was different from other PPI work I’ve been part of. Usually, our involvement is focused and practical shaping the wording of questions, identifying what's meaningful to patients or the public, or suggesting ways findings might be used. XT, however, felt much larger. The issues were harder to "pin down," and in many ways, more emotional. The technology felt both near and distant, near in the sense that real
patients are already receiving pig organs, and distant because the questions it raises are about how we want to live as a society, what kind of science we support, and how we view non-human life. (CMDB)

The wondering involved negotiating the distance between each of us and XT as the object of our attention. Despite XT being a distant prospect for many and a relatively novel idea to the public contributors, some drew on deeply personal perspectives when discussing it. As Isabelle Stengers reminds us, “A problem is always a practical problem, never a universal problem mattering for everybody. Learning is always local” [26]. Public contributors drew on their relationship with science, the world and nonhuman animal species. One public contributor, for instance, shared the story of an aunt who had received a porcine heart valve many years earlier, an intervention that enabled her to live many more years and raise a family. It was this personal connection that sparked their excitement about XT and gave meaning to their involvement in the research.

## XT: disrupting hierarchies

The lack of a settled body of clinical and scientific knowledge of XT meant that, at times, the researchers felt out of their depth, particularly, when public contributors posed questions about the future implementation of the technology. Like the public, the researchers were following the unfolding developments in the US, including recent transplants and plans for clinical trials. Not only that, but at the time of the research there were no cases of XT significantly prolonging life. All patients had died or had the porcine organ removed. The lack of clarity about when or how XT would be implemented meant that, the researchers, too, were left wondering.

**Table:** 

I appreciated that the researchers did not pretend to have all the answers. That honesty allowed us to sit in the uncertainty together. It was both freeing and disorienting. I often found myself wondering not just what I thought about XT, but what kind of person I wanted to be in relation to it. Do I believe that extending a human life justifies ending an animal’s?
If someone in my family needed a transplant, would I feel differently? These are not easy questions, and I don’t think surveys, or even panels like ours, can answer them definitively. But they are questions worth asking. (CMDB)

In this way, XT became something of a disruptive force, unsettling the often-felt hierarchy between professional researchers and public participants in research. In other words, the uncertainty surrounding the field disrupted the usual expert–lay dynamic and fostered a shared sense of curiosity and speculation. Perhaps this was one of the reasons why meetings on XT felt different to meetings held by researchers and public contributors on other projects. The researchers felt that they were not able to provide definitive answers to many of the questions about the future of XT. This, in turn, allowed public contributors to take greater control of the agenda and the structure of the discussions, to introduce new topics and steer the conversation. Rather than merely responding to the practical questions of the survey design, the public contributors scrutinised the scientific developments and scientific narratives of XT in fundamental ways.

At the same time, the public contributors too expressed an awareness of their own limitations. One contributor remarked that the ethical questions raised by XT were “too big for us” and proposed that those who may be more accustomed to grappling with questions of life, death, and the human relationship with other species, such as religious leaders, would be in a better position to approach this topic. This observation resonates with Mike Michael and Nik Brown who, in their discussion on self-representations of XT’s publics, argue that expert and lay discourses may become less distinguishable when members of the public, on the one hand, reflect on their own inadequacies in dealing with the topic and, on the other hand, point out the weakness of the science itself [27].

**Table:** 

We all showed some level of excitement but also a great concern and a sense of responsibility for bringing the topic to the public and taking the debates forward while field of XT is still in its infancy and being shaped. It was nice to see everyone cared so much about such an important topic and that people felt straight away so engaged and lively about thinking it
through. I’ve learnt a great deal from my fellow PPIE members and really value the interesting and enriching discussions we shared. (CL)

## Making XT

As we have already mentioned, most of our projects address policy questions, which, even if innovative, are situated in well-established areas of health and care. In contrast, XT is still an emerging intervention with only a handful of cases being delivered. Because of this emergent status of XT, it becomes more apparent how our work could contribute to the *making* of what XT is becoming. Borup et al. argue that it is highly problematic to assess the “real value” of a technology independently of the expectations projected onto it. They write:

*If we accept that anticipation is actually constitutive of value*,* then we logically cannot differentiate between our expectations of things (biotechnologies*,* stem cells*,* nanotechnologies*,* etc.) and what those things in fact are. Those ‘underlying fundamentals’ are themselves future abstractions*,* expectant projections that alter the now*,* the future working back on the present.* [28]

In other words, expectations and anticipations are not just passive predictions. Instead, they actively shape the development, perception, and value of technologies in the making. In this view, the future is not something that simply arrives later. On the contrary, it influences our actions in the present and, in doing so, is itself shaped by the very expectations projected onto it. In the case of XT, the way in which imagined futures can shape present policy planning was evident in the XT Expert Subgroup’s concerns that public optimism about future access to xenotransplants might reduce willingness to donate human organs, thereby shrinking the pool of available donors.

**Table:** 

I also became more aware of how involvement itself can be complicit in legitimising a controversial technology. By participating, are we helping to make XT more acceptable or more likely? That’s not an easy position to hold, especially when some of us are deeply
ambivalent or even opposed to the technology. It made me realise that involvement isn’t just a technical or democratic exercise, it’s a political and ethical one too. (CMDB)

The public contributors were too concerned about the making of XT. They highlighted how our research was inevitably entangled in the play of expectations and anticipations surrounding XT. For instance, they cautioned that the phrasing of the survey questions could introduce bias by presenting XT in an overly positive light, potentially eliciting more favourable responses. They stressed the importance of maintaining neutrality when presenting the current scientific understanding of XT, in order to avoid fostering positive expectations that could, in turn, help generate a more favourable environment for the emerging intervention. Moreover, a public contributor who argued that XT should be halted altogether, stating that there is no ethical way to pursue it, thought that any research, regardless of its findings, implicitly supports and legitimises its development. This tension revealed how public contributors were not only engaging with the content of the study, but also grappling with the broader implications of being involved in the unfolding of XT. While XT clinical trials have been deemed permissible in the US, its effectiveness and safety remain under investigation, and its implications for UK health policy and healthcare services are yet to be fully understood. As we have shown, public contributors were attentive to the geography of XT innovation, particularly its perceived movement from the US to the UK, which shaped their ethical concerns. In doing so, they incorporated spatial dimensions into their imaginaries of XT futures. This reflects a spatio-material sensibility [29] by pointing out that the location of knowledge production and innovation is not neutral but carries social and ethical significance. In this case, such spatial awareness mobilised an imaginary of the research as a site of caution against the acceptance of XT without adequate scrutiny.

The chosen method, a quantitative questionnaire, was also seen as actively shaping XT by generating knowledge about it, a process that is necessarily partial and incomplete. Some public contributors questioned the interpretability of survey responses, arguing that the complexity of issues we were exploring would be better addressed through qualitative interviews, where follow-up questions could elicit a deeper understanding of respondents’ views (we wished to undertake such interviews after delivering the survey but did not have funding to do so). Concerns were also raised about the accessibility of the survey, which excluded individuals who could not complete it online or by phone. In other words, the public contributors drew the researchers’ attention to the fact that methods do not merely reflect the world but participate in creating it [30]. In the case of a questionnaire on public views of XT, this shaping could happen through potentially omitting significant complexities and nuances in how people relate to or understand the issue.

## Discussion

The uneven power dynamics between researchers and public contributors have been identified previously as a significant challenge to meaningful and ethical public involvement in research [13–15]. It is often argued that clinical or scientific knowledge continues to be privileged over the experiential knowledge that members of the public contribute [13, 31]. To flatten this hierarchy and foster more collaborative working practices, some scholars advocate for dedicated training to develop researchers’ skills to engage with public contributors [13, 32–34].

While we agree that institutional culture change is necessary to support more equitable and less hierarchical forms of collaboration, our reflections suggest that the *object* of the research itself can also shape the dynamics between researchers and public contributors. In our case, XT acted as a disruptive force that unsettled conventional roles and opened up new possibilities for participation for both, researchers and contributors. Specifically, the uncertainty surrounding the field disrupted the usual expert–lay divide as the researchers were unable to answer many of the critical questions about XT’s future. This uncertainty created a space for wondering. It allowed us to move beyond discussions of practicalities, such as wording changes, into more philosophical territory. Questions about the questionnaire’s clarity evolved into broader concerns about how our research might contribute to shaping what XT is or could become. Researchers were cautioned that the act of surveying public opinion was not neutral but potentially legitimising XT as a viable clinical option.

**Table:** 

If there is one thing I would want future projects to take forward from this experience, it’s the importance of creating space for wondering. Not everything needs to be
problem-solved or decided right away. Sometimes, sitting with the discomfort and complexity is what opens up the most meaningful insights, not only for researchers, but for public contributors too. (CMDB)

Moreover, discussions about hierarchies between researchers and public contributors can sometimes inadvertently collapse all researchers and scientists into a single category, overlooking the variation in their expertise and positionality. In our case, researchers engaging with public contributors without assuming expertise in XT was a key part of the dynamic. It was our intention not to invite an XT expert to the meetings in order to support our impartiality as researchers and public contributors as we worked together on the development of the survey and data analysis. As noted earlier, members of the research team who participated in the meetings often felt out of their depth when discussing XT, given the rapidly evolving nature of the scientific evidence at the time. It could be theorised that as they presented themselves as independent researchers without specialist expertise, they created a greater distance between themselves, the scientific community, and the formal policy-making process. As a result, this gave a rise to a space for more candid expressions of discomfort or even disagreement.

It has been argued that there often remains a lack of reflective listening to public concerns and of honest engagement between scientists and society, with the former frequently adopting the view that technology should and will play a dominant role in democratic societies [35]. While we do not claim to offer a universal remedy to this problem, it is often suggested that public engagement is limited at the early stages of research [19]. Our reflections point out that engaging publics in policy research when technologies are still in early stages of development, rather than already routinely adopted in clinical practice, opens up space for those technologies to be questioned, negotiated and resisted. For policy evaluators, such as PIRU, this would mean horizon scanning to identify future policy concerns and inviting public contributors to engage with those concerns before they become immediate.

## Conclusion

Recent advances in XT science have reignited debates about its ethics, safety, and social implications. While scholars have long advocated for the inclusion of the public in cultural and ethical debates concerning XT, we showed how XT itself reshapes public involvement. We argued that XT, a biomedical innovation still under investigation and not yet applied routinely as a viable transplant option, may be a disruptive force within public involvement in policy research. More broadly, biomedical technologies that remain in exploratory phases and less established health care issues may similarly challenge more familiar dynamics of public involvement in policy research. From our collaboration as academics and public contributors, we observed how uncertainty in the field blurred expert–lay boundaries and created space for reflection not typically possible in research designed to meet immediate policy needs. Consequently, what began as practical discussions about questionnaire design opened into broader questions about how research might shape what XT is, or could become.

## Electronic Supplementary Material

Below is the link to the electronic supplementary material.

Supplementary Material 1

## Data Availability

Not applicable.

## Abbreviations

XT: Xenotransplantation
FDA: Food and Drug Administration
ISOU: Implementation Steering Group on Organ Utilisation
DHSC: Department of Health and Social Care
NHS: National Health Service
PIRU: Policy Research Unit in Policy Innovation and Evaluation
NIHR: National Institute for Health and Care Research
PPIE: Patient and Public Involvement and Engagement
