# Supplementary material for: When xenotransplantation enters the conversation: reflections on public involvement in research on a novel and controversial technology
Source: Res Involv Engagem. 2026 May 4;12:55. doi: 10.1186/s40900-026-00894-5 (PMC13137622; doi:10.1186/s40900-026-00894-5)
Supplement: Supplementary file 1 — Supplementary Material 1 [file 40900_2026_894_MOESM1_ESM.docx]

**Appendix 1: Survey questionnaire**

Public contributors have reviewed the survey, advised on wording and co-produced the sections that introduce facts about xenotransplantation (XT). We have highlighted those sections and inserted boxes with text explaining the exact interventions.

MODULE 1: Generalised attitudes to organ donation

**{ASK ALL}**

**XTIntro1**

Now we will ask you some questions on human organ donation and transplantation.

**{ASK ALL}**

**HumanODT**

To what extent do you support or oppose **human organ donation and transplantation**?

INTERVIEWER: READ OUT

1. Strongly support

2. Support

3. Neither support nor oppose

4. Oppose

5. Strongly oppose

**{ASK ALL}**

**ODonate**

Have you thought about donating any of your organs for transplantation at the end of your life?

1. Yes

2. No

**{ASK IF ODonate = 1}**

**ODRWish1**

Have you registered your decision in relation to donating your organs for transplantation, at the end of your life, on the NHS Organ Donor Register?

INTERVIEWER: READ OUT

1. Yes

2. No

3. Can’t remember

**{ASK IF ODRWish1 = 1}**

**ODRWish2**

What was the decision you registered on the NHS Organ Donor Register?

INTERVIEWER: READ OUT

1. I registered my willingness (opt-in) to donate some or all of my organs at the end of my life

2. I registered my unwillingness (opt-out) to donate any of my organs at the end of my life

3. I can’t remember the decision I registered

**{ASK ALL}**

**ODTExp**

Do you, or a family member or close friend, have any experiences of organ donation or transplantation?

1. Yes

2. No

MODULE 2: Religious and moral beliefs

**{ASK ALL}**

**XTRel**

What is your religion?

INTERVIEWER: READ OUT

1. No religion

2. Christian (including Church of England, Catholic, Protestant and all other Christian denominations)

3. Buddhist

4. Hindu

5. Jewish

6. Muslim

7. Sikh

8. Any other religion (please describe)

9. Prefer not to say

**{ASK ALL}**

**RelNFood**

Do you consider yourself to be any of the following?

INTERVIEWER: READ OUT

1. Vegetarian {HL HELPLINK: a person who does not eat meat or fish}

2. Pescatarian [HL HELPLINK: a person who does not eat meat but does eat fish}

3. Vegan {HL HELPLINK: a person who does not eat or use animal products}

4. Mainly vegetarian but occasionally eat meat

5. Someone who eats meat, excluding pork for religious reasons

6. Someone who eats meat

7. None of these

MODULE 3: Attitudes to related scientific and medical issues

**{ASK ALL}**

**GMMeat**

To what extent do you support or oppose **genetically modifying animals to produce meat for human consumption**?

It does not matter if you consume genetically modified meat or not, we just want to know how you feel about it in principle.

INTERVIEWER: READ OUT

1. Strongly support

2. Support

3. Neither support nor oppose

4. Oppose

5. Strongly oppose

**{ASK ALL}**

**AnimalUse**

To what extent do you support or oppose **the use of animals in medical research for human benefit**?

INTERVIEWER: READ OUT

1. Strongly support

2. Support

3. Neither support nor oppose

4. Oppose

5. Strongly oppose

**{ASK ALL}**

**AnimalMaterial**

Human health care has used material derived from animals for many years, including insulin for diabetics, vaccines, and the use of pig heart valves in open heart surgery. To what extent do you support or oppose **the established use of animal tissue in NHS health care**?

INTERVIEWER: READ OUT

1. Strongly support

2. Support

3. Neither support nor oppose

4. Oppose

5. Strongly oppose

MODULE 4: **Prior knowledge of animal-to-human organ transplantation**

**XTIntro**

This questionnaire seeks your views on the transplant of organs (namely the heart and the kidneys) from animals to humans. The use of animal organs could potentially address the shortfall of human organs needed for transplant. However, it may have associated risks. Currently, pig organs are the most likely to be used in animal to human organ transplants.

As this is a new topic, we do not expect you to have prior knowledge. {IF WEB “We will shortly be presenting you with more information – please answer the following questions without checking or referring to any online sources.}

The paragraph introducing XT was co-developed with public contributors.

**{ASK ALL}**

**XTAware1**

Were you aware of the possibility of transplanting animal organs into humans before starting this questionnaire?

1. Yes

2. No

**{ASK ALL}**

**XTReact [MULTICODE]**

What is your gut reaction to the idea of animal to human organ transplants?

WEB: “Please select all that apply”

TEL: “INTERVIEWER: READ OUT EACH OPTION AND CODE ALL THAT APPLY”

1. Surprise
2. Excitement
3. Concern and nervousness
4. It should not be allowed to happen on principle
5. Disgust
6. Uncertainty
7. Other feeling (please describe).

**{ASK ALL}**

**XTReact2**Please tell us why you feel this way.

Including free text responses was strongly suggested by the public contributors who believed that XT was a complex issue and, therefore, required in-depth exploration of the reasoning behind choices made by respondents.

OPEN

MODULE 5: **Attitudes to the transplantation of animal organs into humans**

**{ASK ALL}**

**XAttitudesIntro**

Genetically modified pigs would be specially reared for the purpose of organ donation for transplantation. These pigs would live indoors, in sterile conditions but in social groups. The organs removed from these modified pigs would be screened for known infections before being used for transplant.

The paragraph was co-developed with public contributors.

**{ASK ALL}**

**XTAllow**

Irrespective of whether you would take it up yourself…

Do you believe that animal to human transplantation, as described before, should be allowed?

INTERVIEWER: READ OUT

1. Definitely should be allowed

2. Probably should be allowed

3. Probably should not be allowed

4. Definitely should not be allowed

**{ASK IF XTAllow = 1 or 2}**

**XTAllowReas1 [MULTICODE]**

Why do you think animal to human transplantation **should** be allowed?

WEB: “Please select all that apply”

TEL: “INTERVIEWER: READ OUT EACH OPTION AND CODE ALL THAT APPLY”

1. It will save human lives that would otherwise be lost

2. There is nothing inherently wrong in using animals to help humans

3. It is up to the person receiving the organ to decide what is acceptable to them

4. The person receiving the organ will not be dependent on human donors for a transplant organ

5. Other (please describe)

**{ASK IF XTAllow = 3 or 4}**

**XTAllowReas2 [MULTICODE]**

Why do you think animal to human transplantation **should not** be allowed?

WEB: “Please select all that apply”

TEL: “INTERVIEWER: READ OUT EACH OPTION AND CODE ALL THAT APPLY”

1. It is against my religious beliefs to use pigs for human transplants

2. It is against my moral principles to use animals for human transplants

3. Animals should not be genetically modified solely to provide organs for human transplantation

4. Animals should not be kept in sterile conditions indoors solely to provide organs for human transplantation

5. There are better ways to save the lives of people waiting for organs

6. Other (please describe)

MODULE 6: **Personal acceptability of xenotransplantation and views on risks**

**{ASK ALL}**

**XAcceptIntro**

Kidney and heart transplants using human organs can save lives. However, there is a shortage of suitable human organs for transplants. This scarcity is especially acute for children. Over 7,000 people in the UK are waiting for a transplant, with three people dying each day while waiting.

Although doctors are still some way away from being able to successfully transplant animal organsinto humans, one of the possible risks is the transfer of known or unknown infections. It is also conceivable that close contacts of the patients who have received an animal organ transplant could become infected. This means that it may be necessary to monitor patients and their close contacts at intervals after the transplant.

The paragraph was co-developed with public contributors.

**{ASK ALL}**

**XTAcceptA**

If you needed an organ transplant, would you be willing to accept one from a pig?

1. Yes

2. No

**{ASK ALL}**

**XTAcceptB [MULTICODE]**

In which circumstances, if any, would you be willing to accept a transplant from a pig?

WEB: “Please select all that apply”

TEL: “INTERVIEWER: READ OUT EACH OPTION AND CODE ALL THAT APPLY”

1. If my chances of surviving before a human organ became available were low

2. If I had already had a failed human organ transplant

3. As a temporary measure while waiting for a human organ to become available

4. If I had been told no human organ would be suitable for me

5. If receiving an animal organ would reduce the time I have to wait for a human organ transplant

6. I would be open to having an animal organ in any event as long as it was as effective as a human organ transplant

7. Other (please describe)

8. I would never accept a transplant from a pig [EXCLUSIVE]

**{ASK ALL}**

**XTRecieveA [MULTICODE]**

Which of the following concerns would you have if you were offered a transplant from a pig?

WEB: “Please select all that apply”

TEL: “INTERVIEWER: READ OUT EACH OPTION AND CODE ALL THAT APPLY”

1. My risk of infection from the animal

2. My infection risk to my close contacts

3. My infection risk to the wider public

4. Anxiety that it might not work as well as a human organ, regardless of what I was told by experts

5. If it failed, I might miss my chance to receive a human organ or go down the waiting list

6. None of these concerns [EXCLUSIVE]

**{ASK ALL}**

**XTRecieveB [MULTICODE]**

What other personal concerns would you have, if any, if you were offered a transplant from a pig?

We began with a longer list of potential concerns, public contributors advised us on how to shorten the list for simplicity and to reduce the time required to complete the survey.

WEB: “Please select all that apply”

TEL: “INTERVIEWER: READ OUT EACH OPTION AND CODE ALL THAT APPLY”

1. The consequences to my DNA of mixing animal and human tissue

2. The concern about having to explain to family and friends that I have a pig heart or kidney

3. Knowing when and how to inform any sexual partner(s)

4. How other people might view me (e.g. as ‘less human’)

5. Other (please describe)

6. None of these concerns [EXCLUSIVE]

**{ASK ALL}**

**XTMeasures [MULTICODE]**

If you needed a transplant, what measures would encourage you to consider opting for an animal organ?

WEB: “Please select all that apply”

TEL: “INTERVIEWER: READ OUT EACH OPTION AND CODE ALL THAT APPLY”

1. Approval of the specific organ and procedure by an independent organisation similar to the Medicines and Healthcare products Regulatory Agency (MHRA)

2. A specialist doctor saying it was a suitable treatment for me without excessive risk

3. Leaders in my faith community saying it was allowed for me to receive an animal transplant

4. Ensuring the source animal was treated well during their lifetime

5. Other (please describe)

6. Nothing would ever encourage me to accept an animal organ [EXCLUSIVE]

**{ASK ALL}**

**XTFollowUp**

If you received an animal organ transplant, do you think your close contacts would be willing to be followed up by medical professionals over a long period of time?

INTERVIEWER: READ OUT

1. Most of my close contacts would agree to this

2. Some of my close contacts would agree to this

3. Most of my close contacts would not agree to this

4. None of my close contacts would ever agree to this

MODULE 7: **Treatment priorities, cost and efficacy**

**{ASK ALL}**

**XTWaitList [MULTICODE]**

Who should be invited from the waiting list to take part in early clinical trials designed to find out whether it would be safe and effective to use pig hearts or kidneys for transplantation?

WEB: “Please select all that apply”

TEL: “INTERVIEWER: READ OUT EACH OPTION AND CODE ALL THAT APPLY”

1. Adults

2. Children

3. Healthiest

4. Those who have waited the longest for a suitable organ

5. Those closest to death

6. Those whose previously transplanted human organ has now failed

7. Those who are unsuitable for a human organ

8. Anyone who volunteers

**{ASK ALL}**

**XTPriority2 [MULTICODE]**

If proven safe and effective, which groups, if any should be a high priority to be offered animal organ transplants?

WEB: “Please select all that apply”

TEL: “INTERVIEWER: READ OUT EACH OPTION AND CODE ALL THAT APPLY”

1. Adults

2. Children

3. Healthiest

4. Those who have waited the longest for a suitable organ

5. Those closest to death

6. Those whose previously transplanted human organ has now failed

7. Those who are unsuitable for a human organ

8. Anyone who volunteers

**{ASK ALL}**

**XTPriorityRel**

Some people may have a religious or moral objection to animal organ transplants. If animal to human organ transplants become available on the NHS, do you think these people should be given priority for human organs?

1. Yes

2. No

**{ASK ALL}**

**XTAttitudeB**

When animal organ transplants first become available, it is likely that they will be more expensive than human organ transplants and are unlikely to work as well initially. Over time it is hoped that the costs will drop, and the treatment will become as successful as human organ transplants.

Given this, when should animal organ transplants be made available on the NHS?

The paragraph was co-developed with public contributors.

INTERVIEWER: READ OUT

1. As soon as they become available

2. Only after they become less expensive

3. Only after they become as successful as human organ transplants

4. Only after they become less expensive and as successful as human organ transplants

5. This treatment should not be made available on the NHS at all

**{ASK ALL}**

**XTFuture**

Given that for the foreseeable future, there will still be insufficient human and animal organs for transplantation, should a patient who has already received an animal organ transplant…

INTERVIEWER: READ OUT

1. Be a lower priority for a human organ while their animal organ is functioning satisfactorily?

2. Be an equal priority for a human organ while their animal organ is functioning satisfactorily?

MODULE 8: **Organ donation after death**

**{ASK ALL}**

**XTODD1**

How would the availability of pig organ transplants on the NHS affect your willingness to donate your organs after your death?

INTERVIEWER: READ OUT

1. It would **reduce** my willingness to donate my organs

2. It would **increase** my willingness to donate my organs

3. It would **not affect** my willingness to donate my organs

**{ASK ALL}**

**XTODD2**

How would the availability of pig organ transplants on the NHS affect your attitude to one of your loved ones donating their organs after their death?

INTERVIEWER: READ OUT

1. It would **reduce** my acceptance for them to donate their organs

2. It would **increase** my acceptance for them to donate their organs

3. It would **not affect** my acceptance for them to donate their organs

MODULE 9: **Priority of xenotransplantation over other approaches**

**{ASK ALL}**

**XTPriority [MULTICODE]**

Given that genetically modified pig organs will be expensive should any of the following options receive more funding from the NHS ahead of animal to human organ transplantation?

WEB: “Please select all that apply”

TEL: “INTERVIEWER: READ OUT EACH OPTION AND CODE ALL THAT APPLY”

1. Preventing heart and kidney disease in the first place
2. Increasing the number of human organs available from deceased donors
3. Improving the use of the human organs which are found to be unusable
4. None of the above [EXCLUSIVE]

MODULE 10: **Follow-up research on xenotransplantation**

**{ASK ALL}**

**XTFollowUp**

Have your views on the use of animal organs for transplant in humans changed since you started completing this survey?

1. Yes

2. No

**{ASK IF XTFollowUp = 1}**

**XTFollowUpA**

Which of the following statements best describes your views now on the use of animal organs for transplant in humans?

It does not matter if you would be willing to receive an animal organ transplant or not, we just want to know your views on animal to human organ transplant in principle.

INTERVIEWER: READ OUT

1. Strongly support

2. Support

3. Neither support nor oppose

4. Oppose

5. Strongly oppose

**{ASK ALL}**

**XTFollowUpFuture**

Can we contact you at some point in the future to invite you to take part in further research to understand your views on this topic in greater depth?

1. Yes

2. No
